# Distribution of macular ganglion cell layer thickness in foveal hypoplasia: A new diagnostic criterion for ocular albinism

**DOI:** 10.1371/journal.pone.0224410

**Published:** 2019-11-18

**Authors:** Viktoria C. Brücher, Peter Heiduschka, Ulrike Grenzebach, Nicole Eter, Julia Biermann

**Affiliations:** Dept. of Ophthalmology, University of Muenster Medical Centre, Muenster, Germany; Massachusetts Eye & Ear Infirmary, Harvard Medical School, UNITED STATES

## Abstract

**Background/Aims:**

To analyse the distribution of macular ganglion cell layer thickness (GCLT) in patients with foveal hypoplasia (FH) with or without albinism to obtain new insights into visual pathway anomalies in albinos.

**Methods:**

Patients with FH who presented at our institution between 2013 and 2018 were retrospectively drawn for analysis. Mean GCLT was calculated after automated segmentation of spectral domain-optical coherence tomography (SD-OCT) scans. Patients with FH due to albinism (n = 13, termed ‘albinism FH’) or other kinds (n = 10, termed ‘non-albinism FH’) were compared with control subjects (n = 15). The areas: fovea (central), parafovea (nasal I, temporal I) and perifovea (nasal II, temporal II) along the horizontal meridian were of particular interest. Primary endpoints of this study were the ratios (GCLT-I- and GCLT-II-Quotient) between the GCLT measured in the temporal I or II and nasal I or II areas.

**Results:**

There was a significant difference between the GCLT-I-Quotient of healthy controls and albinism FH (p<0.001), as well as between non-albinism FH and albinism FH (p = 0.004). GCLT-II-Quotient showed significant differences between healthy controls and albinism FH (p<0.001) and between non-albinism FH and albinism FH (p = 0.006). The best measure for distinguishing between non-albinism FH and albinism FH was the calculation of GCLT-II-Quotient (area temporal II divided by area nasal II), indicating albinism at a cut-off of <0.7169. The estimated specificity and sensitivity for this cut-off were 84.6% and 100.0%, respectively. The estimated area under the curve (AUC) was 0.892 [95%CI: 0.743–1.000, p = 0.002].

**Conclusion:**

Macular GCLT-distribution showed a characteristic temporal to central shift in patients with FH due to albinism. Calculation of the GCLT-II-Quotient at a cut-off of <0.7169 presents a new diagnostic criterion for identification of ocular albinism.

## Introduction

Albinism is a hereditary condition of hypopigmentation caused by impaired melanin pigment production. The absence or reduction of melanin has serious implications for the development and maturation of the eye and visual pathway. Thus, individuals with albinism display a variety of characteristic ophthalmic features including reduced visual acuity and stereopsis, translucency of the iris, nystagmus, foveal hypoplasia (FH), and an abnormal decussation pattern at the optic chiasm. However, these clinical signs vary significantly in degree between patients. An abnormal optic nerve fibre decussation with increased contralateral projection of retinal ganglion cells (RGC) from the eye to the brain is the characteristic that is thought to distinguish albinism from other visual disorders resulting in FH. However, this criterion is not fulfilled in any case and the equipment for recording visual evoked potentials (VEP) and in particular the refined albino-VEP paradigm [1] is not universally available. With spectral domain (SD)-optical coherence tomography (OCT), it is possible to visualize the varying degrees of FH that are likely to represent the different stages of arrested foveal development [2].

To study macular structure in yet more detail, new software tools have been introduced for SD-OCT devices such as Heidelberg’s Spectralis (Heidelberg Engineering, Inc., Heidelberg, Germany), allowing each of the macular retinal layers to be identified and measured independently [3,4]. Among other things, the ganglion cell layer thickness (GCLT) can then be calculated in detail, providing helpful supplementary information for the diagnosis of neuro-ophthalmological diseases [5,6]. In normal eyes, GCLT distribution resembles a doughnut-shaped area around an inner annulus [7].

New insights into macular pathophysiology and organisation in FH might be obtained using OCT automated macular segmentation. In routine clinical practice, we noticed a temporal reduction in GCLT and shift of thickness towards the centre in an albino patient. Interestingly, a similar finding was recently reported in a Japanese family with albinism [8]. We therefore decided to retrospectively analyse GCLT distribution in patients referred to our Department of Ophthalmology in Muenster with FH with or without albinism. All data were fully anonymized before they were accessed. Therefore and because this is a retrospective, intra-departmental study the local ethics committee (approval number: 2019-002-f-S) waived the requirement for informed consent. We hypothesised that specific distribution patterns of GCLT occur in different forms of FH.

## Materials and methods

### Patients

Patients with FH who presented at the Department of Ophthalmology, University of Muenster Medical Centre between June 2013 (implementation date of electronic patient record system) and November 2018 were retrospectively drawn for analysis as well as a healthy age-matched control group. A subgrouping was set for FH due to albinism (albinism FH) or FH due to other reasons (non-albinism FH).

The main inclusion criterion was the availability of a macular SD-OCT volume scan to detect and classify the grade of FH and to perform GCLT quantification after automated segmentation. Exclusion criteria were other ocular or neurological pathologies that could affect macular morphology or retinal nerve fibre layer, e.g. age-related macular degeneration, glaucoma and cranial diseases. All patients underwent a standard ophthalmic examination including refraction; best-corrected visual acuity (BCVA), anterior segment examination, fundus examination and SD-OCT scans. Foveal hypoplasia was classified using structural grading described by Thomas et al.[2].

A diagnosis of albinism FH was made, if two of the major criteria (FH grade 3 or more, misrouting in VEP, iris translucency, genetic correlation) together with two minor criteria (fundus hypopigmentation, nystagmus, hypopigmentation of skin and hair) were present, as recently proposed by Kruijt et al. [9]. The aetiologies of FH in patients without albinism could not be sufficiently clarified on the basis of retrospective data. There were no indications of achromatopsia, PAX6 mutations, aniridia, or prematurity in their electronic patient records, thus we assume that the majority of non-albinism FH in our cohort appeared in isolation.

The procedures followed the tenets of the Declaration of Helsinki, and the protocol was approved by the ethics committee of the University of Muenster, Germany.

### OCT imaging and layer segmentation

**Fig 1:**
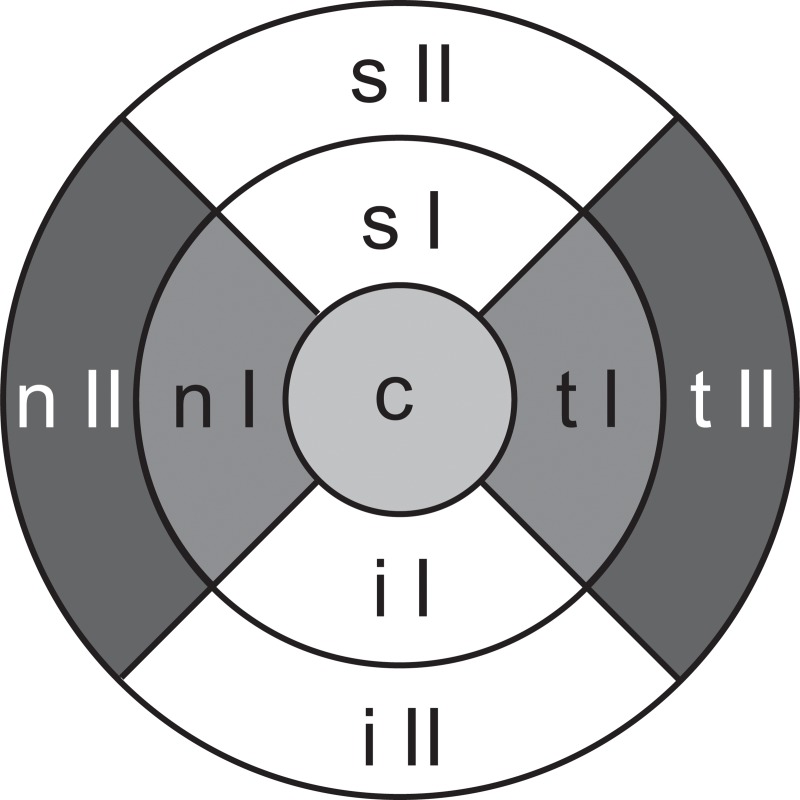
Representative thickness map highlighting the GCLT areas of particular interest along the horizontal meridian with a grey background: central (c, fovea), nasal (n) I and temporal (t) I (parafovea), nasal II and temporal II (perifovea) in a left eye. s: superior, i: inferior.

SD-OCT images were acquired using a Spectralis HRA (Heidelberg Engineering, Heidelberg, Germany), which provides 25 horizontal B-scans (20° x 20° OCT volume scan, 240 μm interscan distance) centred on the fovea with 9 automatic real-time tracking (ART) frames. Segmentation data for different layers within the volume-scan were automatically generated by the integrated software (Eye Explorer Viewing Module 1.0.15.0, Heidelberg Engineering) as described in detail by Invernizzi et al [7]. In brief, this software version provides eight measures: retinal nerve fibre layer (RNFL), ganglion cell layer (GCL), inner plexiform layer (IPL), inner nuclear layer (INL), outer plexiform layer (OPL), outer nuclear layer (ONL), photoreceptors layer (PRC) and retinal pigment epithelium (RPE). Furthermore, the new software automatically provides thickness maps divided into nine subfields in an inner, intermediate, and outer ring with diameters of 1, 3, and 6 mm, respectively, centred on the proposed foveal pit (Fig 1). As visual pathway anomalies along the vertical midline (shifting/misrouting) are a key feature in albino patients, the GCLT areas along the horizontal meridian were of particular interest and used in the analysis. Each image was checked for proper segmentation and proper localisation of the retinal centre and, if necessary, corrected by an expert examiner (VB or JB).

### Statistical analysis

Statistical analyses were performed using IBM^®^ SPSS^®^ Statistics (Version 25, 2017).

To counter the problem of interdependency of the eyes, analyses were performed using the mean of measurements from the left and right eyes of a patient where measurements were taken on both. By taking the mean of both eyes, values tend to be statistically more precise and robust. The study population was described by standard descriptive statistical measures. Continuous variables assumed to be normally distributed are presented as mean ± standard deviation (SD). Non-normally distributed metric variables are reported as median (interquartile range (IQR): 25%quantile– 75%quantile) and categorical variables are shown as absolute and relative frequencies.

Primary endpoints of this study were the ratios (GCLT-I- and GCLT-II-Quotient) between the GCLT measured in the temporal I or II and nasal I or II areas. We analyzed the temporal over nasal thickness expecting to find an essential differentiating criterion between albinism and non-albinism FH due to visual pathway anomalies in albinism. The central area of the fovea shows no pit or is not that reduced in all types of FH-patients (albinism and non-albinism) compared to controls. Thus, the central thickness primarily depends on the degree of FH (Grad 1–4) and not its cause and is therefore of minor importance.

GCLT-I-Quotient = GCLT temporal I / GCLT nasal I

GCLT-II-Quotient = GCLT temporal II / GCLT nasal II

The primary aim of this project was to check whether healthy controls and patients with albinism FH (hypothesis 1 and hypothesis 2) as well as albinism FH and non-albinism FH patients (hypothesis 3 and hypothesis 4) differ in terms of the primary endpoints. Data that were not normally distributed were compared using Mann-Whitney-U-test. In order to control a multiple significance level of 5% in the four tests, the method according to Bonferroni was applied, i.e. each test was carried out at the local level of 5%/4 = 1.25%. All further analyses were exploratory, not confirmatory. A ROC-analysis was performed to find the optimal cut-off (best possible value of GCLT-II-Quotient) for the two-class distinction between albinism and non-albinism (Youden-Index).

## Results

### Clinical data

74 eyes of 38 patients (n = 74) fulfilled the study criteria and were drawn for analyses: 30 eyes of 15 patients (n = 30) of age-matched healthy control subjects, 18 eyes of 10 patients (n = 18) with non-albinism FH (volume SD-OCT scan only unilaterally available in two patients) and 26 eyes of 13 patients (n = 26) with albinism FH. One patient had oculocutaneous albinism, the other patients showed normal pigmentation of skin and hair (ocular albinism). Basic characteristics are listed in Table 1. In our study collective the mean quality score for Non-albinism FH and Albinism FH was comparably well [mean Q 26.6 (from 20–32) versus 28.4 (20–34), respectively.

**Table 1:** Basic characteristics of study population.

	Healthy controls	Non-albinism FH		Albinism FH
n (patients)	15	10		13
Age (years)	20.2 ± 3.17	20.72 ± 14.54		17.30 ± 10.16
Spherical equivalent (Dptr)	-0.55 ± 1.07	-1.14 ± 3.59		0.53 ± 3.71
BCVA (LogMAR)	0.07 ± 0.09	0.38 ± 0.27		0.42 ± 0.25
Major and minor criteria for albinism				
n (eyes)	30	18		26
FH grade 1	0	2		0
FH grade 2	0	11		0
FH grade 3	0	3		23
FH grade 4	0	2		3
Fundus hypopigmentation	0	6		24
Projection abnormality (VEP)	N/A	0*		6*
Iris translucency	0	0		24
Nystagmus	0	4		16
Strabismus	0	4		16
Mean GCLT (μm)				
nasal II	37.93 ± 3.23	31.35 ± 8.10		38.27 ± 4.15
nasal I	50.9 ± 5.24	41.40 ± 15.30		48.58 ± 2.79
central	14.53 ± 3.73	27.75 ± 11.00		38.50 ± 4.67
temporal I	47.1 ± 3.76	36.25 ± 12.34		35.2 ± 3.52
temporal II	35.27 ± 3.05	27.50 ± 5.93		26.92 ± 1.90
p-values				
	Albinism FH—Healthy controls		Albinism FH—Non-albinism FH	
GCLT-I-Quotient	**<0.001**		**0.004**	
GCLT-II-Quotient	**<0.001**		**0.006**	

Basic characteristics of study population, Dptr (Diopter), BCVA (best corrected visual acuity), LogMAR (logarithm of the minimum angle of resolution), FH (foveal hypoplasia), VEP (visual evoked potential), N/A (not available), GCLT (ganglion cell layer thickness), μm (micrometres)

*0 out of 1 and 6 out of 8, statistical significant p-values (< 0.0125) are marked in bold.

Whereas albinism could be reliably diagnosed from the presence of the major and minor criteria listed above, the aetiologies of FH in the 10 patients without albinism could not be sufficiently clarified on the basis of retrospective data. There were no indications for achromatopsia, PAX6 mutations, aniridia, or prematurity in their electronic patient records, thus we assume that the majority of non-albinism FH in our cohort appeared in isolation. The patient with FH grade 4 in the non-albinism group showed normal fundus pigmentation, a normal optic disc and no iris translucency.

### Ganglion cell layer thickness measurements in foveal hypoplasia with and without albinism

**Fig 2:**
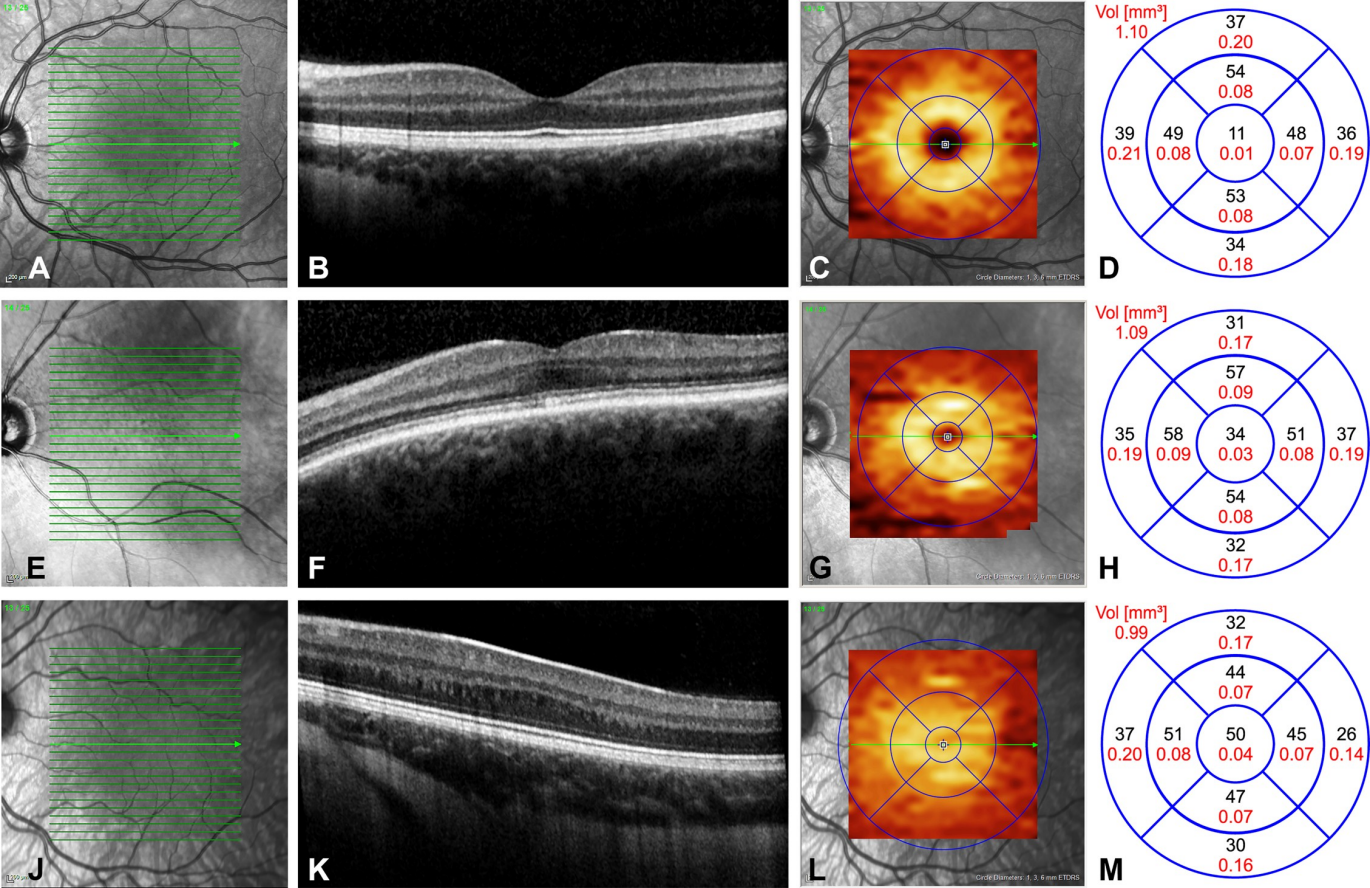
Representative images of left eyes from the three patient groups: A-D healthy controls, E-H non-albinism FH, I-L albinism FH. In healthy controls (Fig 2A–2D), foveal pit formation is detectable through extrusion of the inner retinal layers, ONL widening and lengthening of photoreceptor outer segments (2B). The GCLT distribution pattern forms a doughnut shaped area around an inner annulus (2C). Values of GCLT in μm (black letters) and mm2 (red letters) in the nine areas are given in Fig 2D, showing a circular and homogenous GCLT distribution depending on the eccentricity of the foveal pit with RGC accumulation in the intermediate ring. In a patient with non-albinism FH grade 1 (Fig 2E–2H), the extrusion of the plexiform layers was absent, whereas the other foveal structural features were present (2F). The doughnut shape of the GCLT distribution was still recognizable (2G), although the central area showed markedly higher GCLT values (2H) due to shallower pit formation. In the albinism FH patient (Fig 2I–2L) the fovea could be located only through the widening of the ONL, representing FH grade 3 (2J). The GCLT was markedly reduced in area t II and shifted towards the centre where GCLT values were increased (2L).

In healthy controls, foveal anatomy was regularly in OCT. The GCLT distribution pattern forms a doughnut shaped area around an inner annulus. Values of GCLT in the nine areas are inside the physiological limits, showing a circular and homogenous GCLT distribution depending on the eccentricity of the foveal pit. These findings were homogenously present in all control patients. Representative OCT-pictures of the three study groups are given in Fig 2. In the non-albinism group we observed a more heterogeneous picture of FH from grade 1 to 4 and a nasal and temporal reduction of GCLT. The extrusion of the plexiform layers was always absent, whereas the other foveal structural features were present in relation to FH grading. The doughnut shape of the GCLT distribution was often still recognizable, although the central area showed markedly higher GCLT values due to shallower pit formation. Most of the albinism patients had FH grade 3, less often FH grade 4, which is apparent in the finding of fovea plana in the OCT. Furthermore, a temporal to central shift was detectable in the GCLT analysis. In a representative albinism FH patient (Fig 2I–2L) the fovea could be located only through the widening of the ONL (3J).

**Fig 3:**
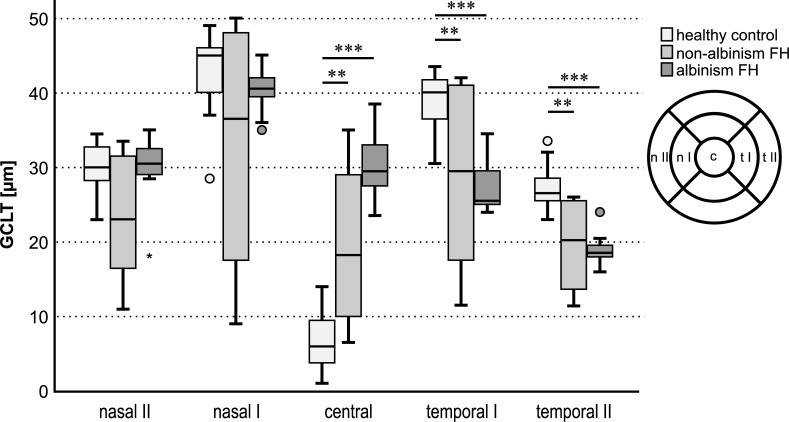
GCL thickness in μm for healthy controls, non-albinism FH and albinism FH in the different areas measured: GCLT nasal II, nasal I, central, temporal I and temporal II.

Macular GCLT-distribution showed a characteristic temporal to central shift in albinism FH. Temporal GCLT was significantly decreased in areas I and II in albinism FH compared to healthy controls. This is evident in the statistical findings below. Fig 3 gives an overview of the GCLT in μm of each of the three groups tested for the five main areas of interest: n II, n I, c, t I, and t II.

Interestingly, the redistribution of the GCLT in the horizontal plain in albinism FH was a phenomenon restricted to the inner retinal layers and declined in significance towards the outer retina (ONL and INL thicknesses of the three study groups are shown in supplemental file A+B).

### GCLT-Quotients differed in albinism FH and non-albinism FH

**Fig 4:**
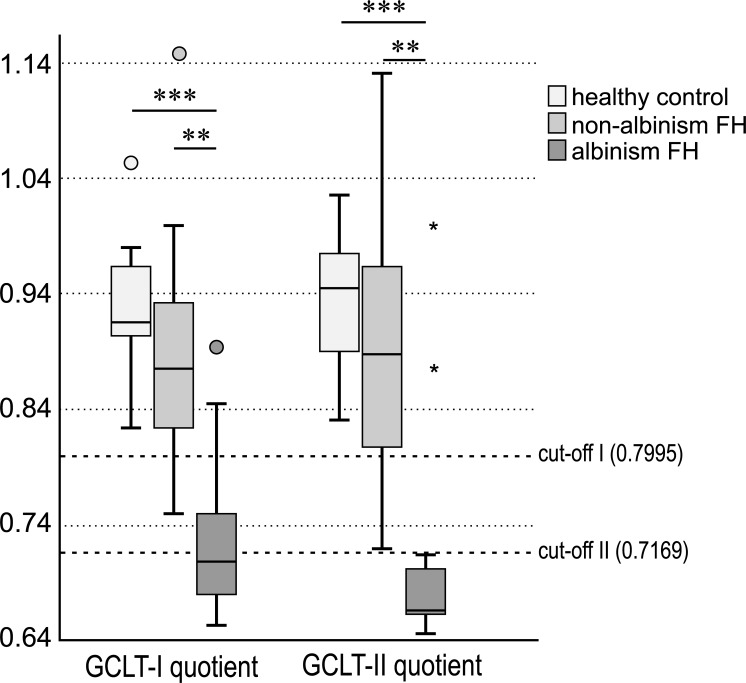
GCLT-I- and GCLT-II-Quotient in healthy controls, non-albinism foveal hypoplasia and albinism FH, significant differences are connected by lines and marked with stars (p< 0.001 = ***; p<0.01 = **). Optimal cut-offs for distinguishing between the albinism FH and non-albinism FH groups for GCLT-I- and GCLT-II-Quotient (0.7995 and 0.7169) are drawn as a reference line to the x-axis.

The median GCLT-I-Quotient was 0.9159 (IQR = interquartile range: 0.9035–0.9667) for healthy controls and 0.8759 (IQR: 0.8234–0.9500) in non-albinism FH while in albinism FH it was 0.7087 (IQR: 0.6766–0.7645). Mann-Whitney-U-test showed a significant difference between the GCLT-I-Quotient of healthy controls and albinism FH (p<0.001), and a further significant difference between non-albinism FH and albinism FH (p = 0.004) (Fig 4). The p-value for the comparison of non-albinism FH and healthy controls was p = 0.196.

The median GCLT-II-Quotient was 0.9452 (IQR = interquartile range: 0.8642–0.9765) for healthy controls, 0.8887 (IQR: 0.7999–0.9695) in non-albinism FH and 0.6667 (IQR: 0.6562–0.7085) in albinism FH. The Mann-Whitney-U-test revealed a significant difference between the GCLT-II-Quotient of healthy controls and albinism FH (p<0.001), and another significant difference between non-albinism FH and albinism FH (p = 0.006) (Fig 4). The p-value for the comparison of non-albinism FH and healthy controls was p = 0.38. Quotients for INL and ONL showed no distinct differences either (Supplemental File C+D).

**Fig 5:**
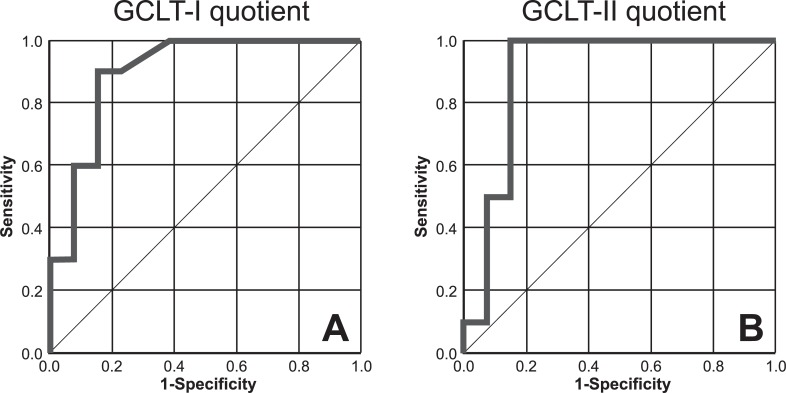
ROC curves, sensitivity and 1-specificity of GCLT-I-Quotient (A) and GCLT -II-Quotient (B) in albinism FH and non-albinism FH.

The optimal cut-off for distinguishing between the albinism FH group and the non-albinism FH group using the GCLT-I-Quotient was 0.7995 (<0.7995: albinism, >0.7995: non-albinism, Fig 4). The estimated specificity and sensitivity for this cut-off were 84.6% and 90.0%, respectively. The estimated area under the curve (AUC) was 0.900 [95%CI: 0.771–1.000, p = 0.001], indicating a marked potential for distinguishing albinism from non-albinism (Fig 5A).

The optimal cut-off for distinguishing between the albinism FH group and the non-albinism FH group using the GCLT-II-Quotient was 0.7169 (<0.7169: albinism, >0.7169: non-albinism, Fig 4). The estimated specificity and sensitivity for this cut-off were 84.6% and 100.0%, respectively. The estimated area under the curve (AUC) was 0.892 [95%CI: 0.743–1.000, p = 0.002], indicating again a marked potential for distinguishing between the presence and absence of albinism (Fig 5B). As the cut-off II shows higher values for sensitivity and consistent values for specificity compared to cut-off I, cut-off II seems to be more predicting.

## Discussion

The key findings of this study are as follows: (1) The OCT GCLT distribution in hypoplastic foveas of albinos showed very specific features in the horizontal plane, with macular ganglion cells displaying a characteristic shift from a temporal to central position. (2) In the non-albinism FH group, GCLT showed a circular peripheral reduction and central increase. (3) Calculation of the GCLT-II-Quotient at a cut-off of <0.7169, presents a new diagnostic criterion for identifying ocular albinism in a patient with FH (specificity 84.6%, sensitivity 100.0%). Our data provides detailed morphometric information on foveal structure in albinism, which could facilitate differential diagnosis of FH.

The ocular manifestations of albinism are multi-faceted, ranging from FH to macular transparency, nystagmus, iris transillumination, strabismus and refractive errors and even to a reversal of the polarity in VEP, as an indicator of optic nerve misrouting. High specificity and sensitivity have been reported for VEP in many clinical trials [1,10,11] but a high inter-individual variability is obtained with this method [10]. Similarly, Kruijt et al. describe a heterogeneous phenotype in albinism, a retrospective cohort study of 522 patients with albinism revealing that 16.1% showed no misrouting in VEP [9]. Within the study cohort, none of the characteristics of albinism were consistently present. The authors therefore introduced major and minor criteria to identify albinism, which we likewise used for diagnosis in our cohort. Nevertheless, some problems remain. Most patients with albinism are reported to have grade 3 FH [2], but FH is also a characteristic feature of PAX6 mutations, achromatopsia, isolated FH [12] and prematurity. Within these entities differentiation can be challenging.

With the GCLT-Quotient, we have introduced a new criterion to distinguish albinism FH and non-albinism FH, as the grading of FH alone was insufficient for this purpose. Visualization of FH with OCT is crucial in guiding the analysis for diagnosis of albinism, especially in patients presenting with less severe clinical manifestations including good visual acuity, absence of nystagmus and non-obvious iris, skin and hair pigmentation [13,14].

SD-OCT is widely used all over the world, and macular segmentation has become a useful tool in the diagnosis of neuro-ophthalmological diseases [5,6]. Our findings may facilitate future diagnosis of albinism in patients with FH, as GCLT distribution offers additional information about its cause. Invernizzi et al [7] recently introduced normative data for retinal-layer thickness maps generated by SD-OCT in a healthy white population. In accordance with Invernizzi et al., we were able to show comparable GCLT values in our healthy controls in all 5 main areas. To the best of our knowledge, OCT GCLT quantification has not yet been systematically addressed in FH. Given that the equipment for recording albino-VEP is not universally available, the analysis of the GCLT-Quotient presents a feasible and non-invasive alternative which, in contrast, is widely applicable.

The distinct pattern of GCLT temporal to the fovea was also recently reported in a Japanese family with albinism [8]. Using a 3D OCT-2000 (Topcon), Oki et al. showed a ganglion cell complex thinning in the temporal region of the fovea, which was noticeably larger in patients with grade 2 than in those with grade 1 FH. Our findings are thus in good accordance with this previous report although the OCT device used and ethnic background of the patients did differ [8]. They found a slight reduction of sensitivity in the nasal visual field, which was not detectable in our cohort and was not a characteristic finding in 15 patients with albinism [15]. Interestingly, Hoffmann et al. detected a specific polarity reversal of the fibres from the central temporal retina by using multifocal VEP [10], whereas at the periphery there was no such polarity reversal, indicating that with increasing eccentricity the projection of the optic nerve reverts to the normal pattern. This may also contribute to preserved visual field sensitivity in albino patients. However, in our experience, reliable visual field testing can be severely hampered in albino patients due to reduced visual acuity, nystagmus, and the young age of the patients, which arguably lowers the validity of this method. Compensatory adaptations according to functional interhemispheric hyperconnectivity of the primary visual areas in albinism may further account for largely preserved visual perception [16] while functional and diffusion-tensor magnetic resonance imaging techniques will hopefully uncover further mechanisms enabling brains of albino patients to functionally adapt to abnormal visual input.

It is tempting to speculate that the reduced GCLT-Quotient in albino patients is either the cause or consequence of the pathogenesis of the misrouting of visual projection. The development of the human fovea occurs from 24 weeks’ gestation to 45 months postpartum [17]. After maturation, the nasal retina projects to the contralateral hemisphere, whereas the temporal retina projects ipsilaterally. Consequently, the line of decussation dividing crossed from uncrossed fibres normally coincides with the vertical meridian through the fovea. This normal projection of ganglion cell axons from the central retina to the brain is severely impaired in FH due to albinism and probably also in other forms of FH. It was assumed that the line of decussation is shifted into the temporal retina in albinism: a great number of fibres from the temporal retina cross the midline at the optic chiasm and project contralaterally [10,18]. This phenotype has been verified in albino animals with an ipsilateral projection and in humans [19,20]. But what if the misrouting of ganglion cell axons is not only a result of a pure guidance defect but also a consequence of a reduction in the number of RGC temporal to the vertical meridian? To address this question, further experiments using OCT segmentation in FH need to be initiated. This investigation is ongoing.

Our study has some limitations worth noting. First, we analyzed a small, retrospective cohort (74 eyes of 38 patients), which however represents the largest FH-cohort in the literature to date with regard to GCLT measurements. Despite a limited sample size, the albino patients described herein exhibit the characteristic phenotypic spectrum of the disease, thus compensating for the missing data concerning molecular DNA analysis.

Another potential point of concern is the inclusion criterion of a volume SD-OCT scan. This represents a selection bias, as patients suffering from severe nystagmus or fixation difficulties were not able to receive a volume SD-OCT and were excluded from examination. However, some of these patients would also be excluded from VEP monitoring.

## Conclusion

Macular GCLT-distribution showed a characteristic central shift and temporal thinning in patients with FH due to albinism. This finding could open up new perspectives for optic nerve fibre projection in albinism. By calculating the GCLT-II-Quotient at a cut-off of <0.7169, a new diagnostic criterion is available to identify ocular albinism as opposed to non-albinism FH.

## Supporting information

S1 Fig ONL and INL thickness in μm for healthy controls, non-albinism FH and albinism FH in the different areas measured: ONL and INL nasal II, nasal I, central, temporal I and temporal II (A,B). ONL-I-, ONL-II-Quotient and INL-I-, INL-II-Quotient in healthy controls, non-albinism foveal hypoplasia and albinism FH, significant differences are connected by lines and marked with stars (p< 0.001 = ***; p<0.01 = **).
(EPS)

## References

[1] Apkarian P, Reits D, Spekreijse H, Van Dorp D. A decisive electrophysiological test for human albinism. Electroencephalogr Clin Neurophysiol 1983;55(5):513–531. 10.1016/0013-4694(83)90162-1 6187545

[2] Thomas MG, Kumar A, Mohammad S, Proudlock FA, Engle EC, Andrews C, et al Structural grading of foveal hypoplasia using spectral-domain optical coherence tomography a predictor of visual acuity? Ophthalmology 2011;118(8):1653–1660. 10.1016/j.ophtha.2011.01.028 21529956 PMC5648335

[3] Garvin MK, Abramoff MD, Wu X, Russell SR, Burns TL, Sonka M. Automated 3-D intraretinal layer segmentation of macular spectral-domain optical coherence tomography images. IEEE Trans Med Imaging 2009;28(9):1436–1447. 10.1109/TMI.2009.2016958 19278927 PMC2911837

[4] Garvin MK, Abramoff MD, Kardon R, Russell SR, Wu X, Sonka M. Intraretinal layer segmentation of macular optical coherence tomography images using optimal 3-D graph search. IEEE Trans Med Imaging 2008;27(10):1495–1505. 10.1109/TMI.2008.923966 18815101 PMC2614384

[5] Cifuentes-Canorea P, Ruiz-Medrano J, Gutierrez-Bonet R, Peña-Garcia P, Saenz-Frances F, Garcia-Feijoo J, et al Analysis of inner and outer retinal layers using spectral domain optical coherence tomography automated segmentation software in ocular hypertensive and glaucoma patients. PLoS One 2018;13(4):e0196112 10.1371/journal.pone.0196112 29672563 PMC5908140

[6] Yamashita T, Miki A, Goto K, Araki S, Takizawa G, Ieki Y et al Retinal Ganglion Cell Atrophy in Homonymous Hemianopia due to Acquired Occipital Lesions Observed Using Cirrus High-Definition-OCT. J Ophthalmol 2016;2016:2394957 10.1155/2016/2394957 27274865 PMC4870342

[7] Invernizzi A, Pellegrini M, Acquistapace A Benatti E, Erba S, Cozzi M, et al Normative Data for Retinal-Layer Thickness Maps Generated by Spectral-Domain OCT in a White Population. Ophthalmology Retina 2018;2(8):808–815.e1. 10.1016/j.oret.2017.12.012 31047534

[8] Oki R, Yamada K, Nakano S, Kimoto K, Yamamoto K, Kondo H, et al A Japanese Family With Autosomal Dominant Oculocutaneous Albinism Type 4. Invest Ophthalmol Vis Sci 2017;58(2):1008–1016. 10.1167/iovs.16-20612 28192564

[9] Kruijt CC, de Wit GC, Bergen AA, Florijn RJ, Schalij-Delfos NE, van Genderen MM. The Phenotypic Spectrum of Albinism. Ophthalmology 2018;125(12):1953–1960. 10.1016/j.ophtha.2018.08.003 30098354

[10] Hoffmann MB, Lorenz B, Morland AB, Schmidtborn LC. Misrouting of the optic nerves in albinism: estimation of the extent with visual evoked potentials. Invest Ophthalmol Vis Sci 2005;46(10):3892–3898. 10.1167/iovs.05-0491 16186379

[11] Coleman J, Sydnor CF, Wolbarsht ML, Bessler M. Abnormal visual pathways in human albinos studied with visually evoked potentials. Exp Neurol 1979;65(3):667–679. 10.1016/0014-4886(79)90052-9 467566

[12] Noval S, Freedman SF, Asrani S, El-Dairi MA. Incidence of fovea plana in normal children. J AAPOS 2014;18(5):471–475. 10.1016/j.jaapos.2014.07.157 25266830

[13] Rossi S, Testa F, Gargiulo A, Di Iorio V, Pierri RB, D'Alterio FM et al The role of optical coherence tomography in an atypical case of oculocutaneous albinism: a case report. Case Rep Ophthalmol 2012;3(1):113–117. 10.1159/000337489 22548044 PMC3339665

[14] McCafferty B, Wilk M, McAllister J, Stepien KE, Dubis AM, Brilliant MH, et al Clinical Insights Into Foveal Morphology in Albinism. J Pediatr Ophthalmol Strabismus. 2015;52(3):167–72. 10.3928/01913913-20150427-06 PMC4948980 26053207

[15] Hoffmann MB, Seufert PS, Schmidtborn LC. Perceptual relevance of abnormal visual field representations: static visual field perimetry in human albinism. Br J Ophthalmol 2007;91(4):509–513. 10.1136/bjo.2006.094854 17372340 PMC1994751

[16] Welton T, Ather S, Proudlock FA, Gottlob I, Dineen RA. Altered whole-brain connectivity in albinism. Hum Brain Mapp 2017;38(2):740–752. 10.1002/hbm.23414 27684406 PMC6866891

[17] Hendrickson AE, Yuodelis C. The morphological development of the human fovea. Ophthalmology 1984;91(6):603–612 10.1016/s0161-6420(84)34247-6 6462623

[18] Prieur DS, Rebsam A. Retinal axon guidance at the midline: Chiasmatic misrouting and consequences. Dev Neurobiol 2017;77(7):844–860. 10.1002/dneu.22473 27907266

[19] Guillery RW, Ombrellaro M, LaMantia AL. The organization of the lateral geniculate nucleus and of the geniculocortical pathway that develops without retinal afferents. Brain Res 1985;352(2):221–233. 10.1016/0165-3806(85)90109-9 4027668

[20] Apkarian P. Chiasmal crossing defects in disorders of binocular vision. Eye (Lond) 1996;10:222–232. 8776452 10.1038/eye.1996.50

